# Knowledge, Attidue and Practice of Advanced Trauma Life Suppoort (ATLS) Protocol among House-Officers in Khartoum State Hospitals, Sudan, 2023

**DOI:** 10.1186/s12909-024-05657-y

**Published:** 2024-06-17

**Authors:** Ola Emad Abdelrahim Mabrouk, Fatima Mohamed Awad Osman, Mustafa Sabir Abakar Awad

**Affiliations:** 1 Faculty of Medicine, Al-Neelain University, Khartoum City, Sudan; 2 Almogtaribeen University, Khartoum City, Sudan

**Keywords:** Trauma, Advanced trauma life support, ATLS, House-officers

## Abstract

**Background:**

Advanced Trauma Life Support was originated mainly to train doctors who don’t manage trauma on a regular basis, including junior doctors as it prepares them more efficiently and effectively for treating and managing trauma patients. This study was conducted to study knowledge, attitude and practice of advanced trauma life support protocol among house-officers in Khartoum state hospitals, Sudan 2023.

**Methods:**

This is a cross-sectional descriptive health facility based study conducted in Bahri Teaching Hospital, Omdurman Teaching Hospital and Ibrahim Malik teaching hospital, Khartoum state, Sudan. Data of 151 House-officers of all nationality working in Khartoum state hospitals was collected using a simple random technique, filling questionnaire that was designed especially for this study. Comparison between different variables by Chi-square test and statistical significance difference at P value < 0.05 was done.

**Result:**

A total of 151 house officers were included in the study. 49% aged between 20 and 25 years, females were the majority 56.3%. About 41.1% have took ATLS course before. 55.21% of the study participants didn’t take the course because it was not available, while 35.42% because it was expensive and 29.17% referred it to their busy lifestyle. 91% of the study population think that ATLS course should be compulsory and 85% think that the ATLS protocol should be recommended to both junior and senior doctors. 77% of the study participants stated that their seniors teaching skills affect how they apply ATLS.

**Conclusion:**

Overall junior doctors at Khartoum state hospitals demonstrated a positive attitude towards ATLS, but they showed poor knowledge regarding the topic. It’s advised that an earlier training program is introduced by incorporating ATLS course to be part of all final year medical school’s curriculum.

## Introduction

Trauma is a driving cause of death worldwide (12% mortality rate) [1]. Yearly, over 100 million people are handicapped temporarily or permanently due to trauma [2], increasing socioeconomic losses, medical care expenditures and disability rates [3], especially in low and middle income countries where trauma accounts for 90% of all injury-related deaths [4].

Advanced trauma life support (ATLS) is a training program for physicians to handle acute traumas [5] and intensive injuries [6], by teaching clinicians a single dependable approach [7] for a quick and precise evaluation of patient’s physiological status and follow-up [5], with hands on procedures for the management [6]. Especially those who don’t treat trauma regularly including junior doctors, and where there may only be one doctor and one nurse available [8]. It emphasizes a systematic approach to trauma care that helps optimizing the use of healthcare resources [9].

It was formulated then adopted by the American College of surgeons Committee on Trauma in 1976 [6]. Firstly introduced in Nebraska in 1978 and reached North America in 1980s [10]. By the year 2000, 13,000 doctors had been trained in 750 courses around the world [11]. It was executed in more than 60 nations around the world [12]. Now it’s acknowledged as a standard of care for the first (golden) hour in trauma centers worldwide [9] (as 30% of all in-hospital trauma death happen within the first hour) [7]. The course is divided to cognitive domain (knowledge base) and trauma management skills (maintain priority and order) [13] and it includes simulate clinical practices, seminars, group meetings, and stations for practical skills [14].

Doctors who completed ATLS course or a shorter version of it were more efficient in managing simulated trauma cases [15] and Studies show that teaching advanced trauma life support course to medical students results in significant improve in knowledge and skill [14].

It is crucial to develop and implement such courses like the Advanced Trauma Life Support course, mentioning that the acquired knowledge and skills begin to deteriorate steadily six months following the course [16]. Cognitive skills deteriorate faster than practical skills [4]. In low trauma volume populations, doctors’ clinical skills decreased after two years, compared to those working in high trauma volume environment—because they have ongoing maintenance of skills— [17]. And in 1992 at Trinidad and Tobago, the majority of road traffic accident deaths occurred when junior doctors and house officers were on duty, so this course is directed to help them improve their trauma care [18]. And Ann R Coll in 2008 proposed that ATLS training should be mandatory during the first foundation year [19]. However, high start-up costs limits the widespread learning of the course in low-income countries [14].

Without proper ATLS training, healthcare providers may struggle to manage trauma cases effectively, leading to higher mortality rates [9]. Lacking knowledge and practice in ATLS may also result in incorrect assessment, increasing severity of injuries and complicating the recovery process [9].

Overall, the appropriate practice of ATLS is essential for enhancing the survival and outcomes of trauma patients and maintaining the resilience of communities in the face of traumatic events. And more studies needs to be done in assessing the level of knowledge and the attitude towards advanced trauma life support protocol among junior doctors and identify limitations regarding this. This study focused on assessing the knowledge, attitude and practice of Advanced Trauma Life Support protocol among house officers working in Khartoum state hospitals.

## Research methods

### Study design/setting

A descriptive cross sectional health-facility based study. It was conducted in Khartoum state which is the capital city of Sudan, it consists of three major localities (Bahri, Southern Khartoum and Omdurman). The study took place in the state’s three major public hospitals Bahri teaching hospital which covers the entire Bahri locality, Ibrahim Malik teaching hospital which covers southern Khartoum locality, and Omdurman teaching hospital which covers Omdurman locality.

### Study population

House-officers (junior doctors) of all nationality both genders who worked in the selected hospitals were included in the study. All house-officers working in private hospitals that have accidents and emergency room in it were excluded.

### Study variables

Dependent variables: Knowledge, attitude and practice of ATLS among house-officers.

Independent variables: Age, gender, Frequently managing trauma patients, ATLS course completion, work in accidents and emergency room, application of ATLS protocol, senior teaching skills.

### Data collection tools and methods

The data was collected using a pretested, structured, close-ended questionnaire specially designed for this study, after ensuring that it conforms to the approved scientific condition. A face-to-face interview was done with each participant as a method of data collection.

### Sample size and sampling technique

The population under the study were house officers in Bahri, Ibrahim Malik and Omdurman teaching Hospitals, at the time of the study. We used the Population formula: n = N/ (1 + N*D^2^), where n = sample size N = population size (244), D = margin of error (0.05) hence the calculated sample size was 151 house officer, all of the sample size was obtained. The sampling method was simple random sampling technique as the data collection process took place each other day in the selected hospitals.

### Data analysis

Data were cleaned using Microsoft Excel® 2016 to ensure integrity by deleting duplications, correcting spelling mistakes and fill out or delete empty fields. After the data were cleansed and a Statistical Package for Social Sciences (V23) was used for data analysis. The data analysis plan included: Chi-square test, descriptive statistics and summary measures, data displayed in tables and figures. The statistical significance level was set to a p-value < 0.05 was considered statistically significant.

## Results

A data of 151 participants were collected for this study. The largest age groups in the study population were those 20 to 25 years old (49%), followed by those 25 to 30 years old (46.4%), the smallest age group were those over 30 years old (4.6%). While the majority of the study population were females (56.3%), and 43.7% were males. 41.1% of the study population took the ATLS course before, while 58.9% did not, as shown in (Table 1).

The participants were asked to rate their adequacy in ten areas, listed in (Table 2) measure their knowledge and practice of the ATLS protocol, the participants were asked to rate their current adequacy as good, average, or bad in the ten areas, and chi square test was used to test for significant association (P value < 0.05) between any of the ten areas and attending the ATLS course, working in the emergency room, frequency of managing trauma cases and good senior teaching skills as the main factors that affect knowledge and practice of the participants. Other factors that affected knowledge and practice as perceived by the study participants are listed in (Table 3).

**Table 1:** Demographic characteristics of study participants

Demographic variable	Frequency	Percentage
Age		
20–25	70	49%
25–30	74	46.4%
More than 30	7	4.6%
**Gender**		
Male	66	43.7%
Female	85	56.3%
**Did you took ATLS course before?**		
Yes	62	41.1%
No	89	58.9%
Total	151	100%

**Table 2:** Shows a summary of the participants repossesses to the ten areas

Correlation between attending the ATLS course and	Attended the ATLS course			Did not attended the ATLS course		
	Good	Average	Bad	Good	Average	Bad
Handling patients when arriving to ER	62.5%	35.9%	1.6%	44.8%	45.85	9.4%
Handle airway and maintain breathing	65.6%	28.1%	6.3%	39.6%	46.9%	13.5%
Shock assessment and resuscitation	64.1%	29.7%	6.3%	46.9%	36.5%	16.7%
Handle head or spinal cord injury	39.1%	40.6%	20.3%	17.7%	46.9%	35.4%
Handle and treat burns	43.8%	35.9%	20.3%	23.0%	36.5%	38.5%
Handle patients with abdominal or pelvic trauma	34.4%	50.0%	15.6%	19.8%	51.0%	29.2%
Handle patients with thoracic trauma	40.6%	51.6%	7.8%	26.0%	39.6%	34.4%
Assessment of Glasgow coma scale	78.1%	18.8%	3.1%	49.0%	41.7%	9.4%
Work according to ABCD approach	84.4%	15.6%	0%	65.6%	26.0%	8.4%
Interaction with trauma team	73.4	25.0	1.6%	45.8%	42.8%	11.5%

**Table 3:** Other factors that affect the application of the ATLS protocol as perceived by the study participants

Factors affecting the application of the ATLS protocol	Frequency	Percentage
Availability of seniors and equipment	1	8.33%
Books, or textbooks	2	16.66%
Facilities	1	8.33%
Good facilities, organized team work, senior presence	1	8.33%
Frequent updates and workshops and continuous practice.	1	8.33%
Number of patients	1	8.33%
Seniors must always check if I’m doing the maneuvers right or not plus having a regular skills training would be beneficial.	1	8.33%
The availability of assessment equipment in the emergency department	1	8.33%
University	1	8.33%
Workshops and Textbooks	1	8.33%
Yes, Hospital contribution to Trauma management courses	1	8.33%
Total	12	100%

Attending the ATLS course was the most significant factor that affected the self-assessment of the participants in the ten areas, there was a significant association in all ten areas with taking the course, and a bivariate analysis revealed a significant positive correlation between taking the course and a good assessment in all ten areas (Table 4).

**Table 4:** Bivariate analysis showing the correlation between taking the ATLS course and the areas assessed

Correlation between attending the ATLS course and	Correlation coefficient (Pearson *R*)	*P* value
Handling patients when arriving to ER	0.205	0.009
Handle airway and maintain breathing	0.243	0.002
Shock assessment and resuscitation	0.193	0.015
Handle head or spinal cord injury	0.240	0.002
Handle and treat burns	0.227	0.004
Handle patients with abdominal or pelvic trauma	0.196	0.013
Handle patients with thoracic trauma	0.272	0.001
Assessment of Glasgow coma scale	0.279	0.000
Work according to ABCD approach	0.236	0.003
Interaction with trauma team	0.290	0.000

31.95 of the study population always works in the emergency room, 45.6% sometimes work in the emergency room, 12.5% rarely works there, and 10% never do. The study showed that working in the Emergency department was associated significantly with the majority of the self-assessment areas in both groups (those who have attended the ATLS course and those who did not) (Table 5).

**Table 5:** The association between working in the Emergency department and the areas assessed in both groups

Association between Working in the Emergency and:	Not attended	Attended
	P value	P value
Handling patients when arriving to ER	0.001*	0.001*
Handle airway and maintain breathing	0.000*	0.2
Shock assessment and resuscitation	0.020*	0.000*
Handle head or spinal cord injury	0.436	0.094
Handle and treat burns	0.1	0.016*
Handle patients with abdominal or pelvic trauma	0.042*	0.226
Handle patients with thoracic trauma	0.231	0.015*
Assessment of Glasgow coma scale	0.533	0.031*
Work according to ABCD approach	0.032*	0.003*
Interaction with trauma team	0.003*	0.1

* Statistically significant (P value < 0.05)

Only 13.1% of the study population never manages trauma patients (*n* = 21), 20.6% always manages trauma patients, 50.6% sometimes manages trauma patients, and 15.6% do so rarely. Of those who manage trauma cases (*n* = 139), 79% (*n* = 110) applies the ATLS protocol, while 21% (*n* = 29) do not. Interestingly while the frequency of managing trauma patients was significantly associated with the assessment in 7 of the 10 areas questioned in the group that attended the ATLS course it showed no significant effect in all ten areas in those who have not taken the course, as shown in (Table 6).

**Table 6:** The association between frequency of managing trauma patients and the areas assessed in both groups

Association between frequency of managing trauma patients course and	Not attended	Attended
	P value	P value
Handling patients when arriving to ER	0.110	0.000*
Handle airway and maintain breathing	0.076	0.326
Shock assessment and resuscitation	0.326	0.002*
Handle head or spinal cord injury	0.728	0.284
Handle and treat burns	0.143	0.006*
Handle patients with abdominal or pelvic trauma	0.415	0.029*
Handle patients with thoracic trauma	0.168	0.010*
Assessment of Glasgow coma scale	0.527	0.207
Work according to ABCD approach	0.144	0.029*
Interaction with trauma team	0.272	0.000*

*Statistically significant (P value < 0.05)

77% of the study population say that their seniors teaching skills affect how they apply ATLS, while 23% disagree. The result showed that having a senior who is good at teaching was significantly associated with only three out of the ten self-assessment areas in those who did not attend the ATLS course, but it was associated with seven out of the ten self-assessment areas in those who did attend the course (Table 7).

**Table 7:** The association between having a senior who is good at teaching and the areas assessed in both groups

Association between having a senior who is good at teaching and	Attended	Not attended
	P value	P value
Handling patients when arriving to ER	0.005*	0.006*
Handle airway and maintain breathing	0.266	0.068
Shock assessment and resuscitation	0.003*	0.349
Handle head or spinal cord injury	0.412	0.349
Handle and treat burns	0.039*	0.029*
Handle patients with abdominal or pelvic trauma	0.003*	0.008*
Handle patients with thoracic trauma	0.005*	0.164
Assessment of Glasgow coma scale	0.221	0.24
Work according to ABCD approach	0.005*	0.078
Interaction with trauma team	0.004*	0.165

*Statistically significant (P value < 0.05)

### Attitude

91% of the study population think that ATLS course should be compulsory, while 9% don’t. 66.3% of the study population think that the ATLS course is essential for doctors whose specialty doesn’t involve regular management of trauma cases, 27.5% think that it has some value for these doctors, and 6.3% think it is completely unnecessary for such doctors. 57.8% of those in the study participants who took the ATLS course say it greatly improved their application of the ATLS protocol, 40.6% of those who took it said it improved their application of the ATLS protocol to some extent, and only 1.6% say that it did not improve their application of the ATLS protocol at all. The majority of the study population, 85% think that the ATLS protocol should be recommended to both junior and senior doctors, 7.5% think it should be recommended to junior doctors, 6.9% think it should be recommended to senior doctors, and 0.6% think it should be recommended to postgraduate medical students and nurses as well as senior students.

Limitations in conducting ATLS course as described by the participants, listed in (Table 8).

**Table 8:** Shows limitations in taking ATLS course as perceived by the study participants

The participants’ reasons for not taking the ATLS course	Frequency	Percentage
Not available	53	55.21%
Expensive	34	35.42%
Busy lifestyle	28	29.17%
It’s not necessary	6	6.25%
I couldn’t find enough time for it	1	1.04%
I don’t know where to take it	1	1.04%

## Discussion

ATLS has been considered as very successful as conducted by course evaluation feedback, participation of physicians and the support of medical-educational institutions worldwide [20]. As in low and middle income countries, lack of skills of healthcare providers may contribute to the compromised outcomes of injury, and short courses can be a very useful solution for skill-deficits [21]. In a study conducted by Koorosh Ahmadi on the effect of ATLS program on medical interns’ performance in simulated trauma management, they showed that there is statistically significant increase in interns’ clinical knowledge and skill performance when comparing between them before and after taking the course [22]. Also in our study, attending ATLS course was the most significant factor that affected the self-assessment of the participants in the ten areas with a significant positive correlation between taking the course and a good assessment in all ten areas. Moreover, when we asked the participants who took the ATLS course about their improvement in applying ATLS protocol, the majority stated that they had greatly improved. In a study conducted by Jameel Ali assessing the effect of trauma volume on skills attrition among physicians completing ATLS course, showed that at 2 years after ATLS the high trauma patient volume group maintained higher MCQ scores than those with the low trauma patient volume [17]. In our study, we suggest that managing more trauma patients without taking the course do not increase the self-perceived competence of the participants (Table 4). In addition, working in the Emergency department improves self-perceived competence regardless of taking the ATLS course or not as seen in (Table 3), which is a somewhat expected finding as practice makes perfect and in this case practice improves practice. This study also found that having a senior with good teaching skills is a lot more useful for those in the study population who have completed the ATLS course compared to those who did not (Table 5).

In our study, we considered that generally, attitudes towards ATLS were positive. In a similar study conducted in Ethiopia showed that among medical intern’s respondents, the majority of them thought that all existing consultants dealing with trauma patients should have done an ATLS course (92.4%), then (76.2%) thought that ATLS course is essential for doctors who do not manage trauma on a regular basis. (19.2%) thought that it offers major advantage, (4.2%) thought it is unnecessary with some advantage and only (0.6%) thought it is completely unnecessary [23]. While the majority in our study thinks that the ATLS course is essential, especially for doctors who don’t manage trauma on regular basis and (91%) of participants thought it should be compulsory.

A study found that funding problems for the course had been experienced by (14%) of trainees [24]. Another study done in 2015 by Payam Tanghi, stating that although (80%) of respondents felt that ATLS would meet their educational needs, they indicated difficulty in accessing the course, and thus suggesting that limitations in accessibility may explain why one-third of physicians do not have recent ATLS certification [25]. In our study, when those who did not take ATLS course (*n* = 96) were asked about their limitations in conducting the course, (55.21%) of them said it is because the course was not available, while (35.42%) say that it is because it was expensive and (29.17%) said it was because of their busy lifestyles, which all constitutes obstacles that could easily be mitigated through the ministry of health or the hospital’s administration by providing a yearly or semi-annually ATLS course for all doctors at the hospital with a focus in new interns.

## Conclusion

The study sheds light on the importance of proper ATLS training among junior doctors. Overall junior doctors at Khartoum state hospitals demonstrated a positive attitude towards ATLS, but they showed poor knowledge regarding the topic, the majority of the participants indicated that most of their knowledge is attained from their seniors upon the clinical practice rather than proper prior training, which is indicative of lack of proper curricula in medical schools and the absence of pre-internship preparatory course.

### Recommendations

- We recommend a larger study sample and an objective assessment method rather than the subjective one.
- Introduction of ATLS course as part of all final year medical schools’ curriculum.
- Governmental and stakeholders support in ATLS funding to insure wider coverage of trainees with regular and organized training.

### Study limitations

- The study population was relatively small.
- The study was conducted in only in Khartoum state.
- Biases that come along using subjective assessment tool.

## Data Availability

All data generated or analysed during this study are included in this published article.

## Abbreviations

ATLS: Advanced Trauma Life Support
